# Enhancing interprofessional education readiness in undergraduate dental students: a scenario-based peer learning programme

**DOI:** 10.1186/s12903-024-03878-7

**Published:** 2024-01-22

**Authors:** Gül Çelik, Ömer Faruk Sönmez, Aysel Başer

**Affiliations:** 1 Faculty of Dentistry, Department of Endodontics, Izmir Democracy University, Izmir, Türkiye; 2 School of Medicine and Population Health, University of Sheffield, Sheffield, UK; 3 Faculty of Medicine, Department of Medical Education, Izmir Democracy University, Izmir, Türkiye

**Keywords:** Interprofessional education, Scenario-based peer training, Dental education, Professionalism, Teamwork, Professional roles

## Abstract

**Background:**

Interprofessional Education (IPE) is an educational approach that brings together students from different healthcare professions to foster collaborative learning and teamwork. Before integrating IPE into the curriculum of health preprofessional students, it is necessary to increase their readiness for IPE. Dentistry increasingly values interprofessional collaboration and teamwork for enhanced patient care and healthcare team competencies, an emphasis also echoed by recent dental education authorities. The aim of this quasi-experimental research was to assess the influence of Scenario Based Learning Peer Learning (SBPL) programme, which involved scenarios necessitating interprofessional communication, on the readiness for IPE among a cohort of undergraduate dental students studying within the framework of the European Higher Education Area (EHEA).

**Methods:**

This study investigates undergraduate dental students’ readiness for IPE and the influence of SBPL programme on their readiness. Participants (*n* = 25) from 18 EHEA countries completed the Readiness for Interprofessional Learning Scale (RIPLS) before and after SBPL programme, held at the 70th European Dental Students’ Association (EDSA) meeting. Data were analyzed using the Wilcoxon Signed Rank Test (*p* = 0.05).

**Results:**

After the SBPT programme, there was a statistically significant increase (*p* < 0.05) in the mean of the total scale, teamwork and collaboration, roles and responsibilities and professional identity subscale. In general, SBPL programme showed a constructive effect on interprofessional readiness. Although there was no statistically significant increase only in items 9,12,18 of the 19 items of the RIPLS, there was an increase in the averages in all except item 12.

**Conclusion:**

Our research emphasizes the importance of diverse perspectives and IPE in the realm of dental education. Within the limits of this study, it showcases the efficacy of a brief half-day SBPL programme with interprofessional scenarios in enhancing participants’ readiness. The programme notably enhanced dental students’ readiness in grasping crucial aspects of IPE: teamwork and collaboration, professional identity, and roles and responsibilities. However, this study does not delve into the potential impact of a comprehensive, long-term curriculum integrating IPE principles. This gap underscores the need for further exploration into the sustained influence of IPE on the interprofessional skills of dental school graduates.

**Supplementary Information:**

The online version contains supplementary material available at 10.1186/s12903-024-03878-7.

## Background

Given the intricate and multifaceted nature of patients’ health needs, it is imperative for diverse healthcare professionals to collaborate, combining their respective knowledge, skills and perspectives, towards a unified and comprehensive approach [1, 2].

Of note is that Field et al. (2023) have documented a position paper stemming from the O-Health-Edu project, which endeavors to gain a deeper understanding of the prevailing state of education among Oral Health Professionals (OHPs) in Europe and to forge a unified vision for this educational domain [3]. O-Health-Edu’s viewpoint regarding the education of OHPs in Europe aligns with the set milestones [4], resolutions [5], and outlined goals of the EU4Health initiative [6] forecasted for the upcoming 20 years until 2040. This inclusive perspective takes into account different stakeholders to guarantee that the education of OHPs contributes to the welfare of both students and patients, seamlessly integrating within a wider healthcare structure. Remarkably, this stance underscores the importance of interprofessional education (IPE), alongside various pivotal educational components.

Interprofessional collaboration refers to the concerted efforts of health professionals from different disciplines in collaboration with patients, caregivers, families, and community members, to provide high- quality health services [7]. IPE is defined as “students of two or more professions associated with health or social care, engaged in learning with, from, and about each other.” The " traditional " definition of IPE, certainly in clinical contexts, is about how we teach students to take a patient-centred view and to bring together the insights of different health professionals in order to treat that patient [8].

Interprofessional education has been gaining recognition as an innovative approach to improve health outcomes in dental education [9] IPE also plays a critical role in addressing the patients with special needs [10, 11], eliminating health inequalities [7], utilizing the resources effectively [12], improving the skills and knowledge of the health care team [13], preventing and addressing associated diseases [14], connecting general and oral health [9, 15]. Some challenges associated with IPE were also defined, such as lack of student motivation, need for additional human resources and time [11, 16].

One of the key aspects of IPE is its encouragement of active learning styles. These methods encompass educational approaches such as scenario-based learning, peer learning, and problem-based learning [17]. Scenario-based peer learning (SBPL) programme helps students enhance their abilities to analyze situations and generate solutions [18]. Peer learning offers students the chance to learn from their peers and engage in collaborative efforts [19].

The integration of IPE into dental education is aimed at improving the quality of education and achieving fundamental program competencies, including the provision of “comprehensive healthcare services” [20]. Readiness for IPE helps facilitate the acquisition of various learning outcomes, such as ethical awareness, understanding of roles and responsibilities, effective communication and collaboration, the development of teamwork and leadership skills, the adoption of a multidisciplinary perspective, analytical thinking, instilling a lifelong learning mentality, practicing evidence-based methods, and embracing innovations [20]. Consequently, the primary objective is to empower dental students to enhance their interprofessional collaboration and communication skills, enabling them to deliver patient-centered and comprehensive healthcare services [21].

In 2017, during the collaborative assembly of the American Dental Education Association (ADAE) and the European Dental Education Association (ADEE) in London, a consensus emerged asserting the imperative transition from the conceptualisation interprofessional education to its practical implementation [22]. This significant resolution gained further momentum and reinforcement at the symposium titled “ADEE, ADAE Shape the Future of Dental Education” [23] held for the third time in 2019.

The directives articulated by these authoritative bodies at both regional and national levels, in the field of dental education have precipitated a critical examination of the level of student readiness and the strategies required to enhance this readiness. Within the framework of our research design, these prompts from the educational leadership have wielded discernible influence across various facets, encompassing the identification of the specific target demographic, the selection of a pertinent assessment instrument, and the formulation of a SBPL milieu. The hypothesis of this study is that the SBPL programme enhances undergraduate dental students’ readiness for IPE.

The aim of this quasi-experimental research was to assess the influence of SBPL programme, which involved scenarios necessitating interprofessional communication, on the readiness for IPE among a cohort of undergraduate dental students studying within the framework of the European Higher Education Area (EHEA).

## Materials and methods

### Participant demographics and data collection

A cohort of more than 120 students from institutions aligned with the EHEA convened at the 70th Annual Meeting of the European Dental Students’ Association (EDSA) in Palma De Mallorca, Spain, held from the 20 to the 27 of August. These participants, who constituted the target population, were invited to participate in the study. Ultimately, a voluntary assembly of 25 students granted their informed consent and thus participated (*n* = 25). Notably, the composition of participants spanned senior cohorts encompassing of the 3rd, 4th, 5th, and 6th years, with the exception of one participant from the 2nd year.

The EDSA congress, held biannually, garners significant attention from European students. Due to logistical constraints, not all applicants can participate. The EDSA board selects 120 students based on criteria like gender, country, class, previous congress attendance, and motivation via quota sampling. Selection made by EDSA board is blind, with names concealed. Eligible 120 students of the congress receive the congress program and are asked to register for sessions. SBPL programme is one of the sessions on the congress agenda that they participants may register. SBPL programme received 34 registries and out of 34 students opting for SBPL programme, 25 attended in the programme in two rounds.

The data collection process was carried out as a two-stage approach including pre and post SBPL programme evaluations in the form of a questionnaire consisting of scale and sociodemographic data. Participants completed the pre-questionnaire on their personal mobile devices via Google Form using the distributed QR code within the prescribed 20 min. Following the SBPL programme, a different QR code was distributed to the participants and they again completed the post-questionnaire via Google Forms without any problems.

Within the questionnaire, participants were prompted to provide pseudonyms as well as pertinent demographic details encompassing age, gender, and institutional affiliations. This facilitated pre- and post-scale matching for subsequent analysis. Sample size was determined using G*Power 3.1.7 software. Considering the pre-SBPL programme mean score of 46.30 ± 1.30 and the post-SBPT mean score of 27.44 ± 1.30, the “effect size” (d = 0.6) was ascertained, thereby deducing the requirement for a total of 32 volunteers to achieve an 80% statistical power at a significance level of *p* = 0.05.

### The readiness for interprofessional learning scale

The RIPLS, developed by Parsell and Bligh and subsequently refined by McFadyen, Webster, and Maclaren, is a multi-dimensional assessment tool consisting of three distinct sub-dimensions [14]. It is employed to quantitatively evaluate participants’ inclinations and perspectives regarding collaborative teamwork and interprofessional cooperation (Items 1–9), as well as their notions of professional identity, encompassing both negative (Items 10–12) and positive (Items 13–16) dimensions. The scale also measures attitudes towards roles and responsibilities (Items 17–19).

The RIPLS, a rigorously validated and dependable instrument, comprises 19 individual items that are appraised scored on a 5-point Likert scale, wherein the responses range from “Strongly Disagree” (rated as 1) to “Strongly Agree” (rated as 5). Specifically, the professional identity component is dissected into two sub-dimensions: the domain of negative professional identity and that of positive professional identity. Items 10, 11, and 12 of the negative professional identity sub-dimension pertain to preconceived notions and biases, and their scoring was reversed during data analysis (e.g., “Strongly Disagree” scored as 5, “Strongly Agree” scored as 1) [15].

### Scenario based peer learning progamme

SBPL programme was implemented with the aim of enhancing interprofessional education readiness among dental students by utilizing scenarios that emphasize interprofessional collaboration, teamwork, and roles-responsibilities (see the format in the Supplementary Material 1). The session, conducted on August 22, 2022, involved 25 participants from various dental school years, guided by peer trainers from diverse healthcare backgrounds. The SBPL session had a structured format, including an introductory segment (15 min.), an icebreaker activity (5 min.), small group interactions centered around scenarios (40 min), group presentations (20 min), Q&A and Group Discussion (15 min) and conclusion and Debrief (10 min).

In the implemented scenario-based methodology, participants underwent an initial phase of introduction (15 min) and orientation through a 20-minute presentation. This presentation aimed to furnish participants with illustrative instances delineating the extent and significance of IPE along with its concomitant collaboration aspects. Following this presentation, participants were subsequently grouped into clusters of five individuals each, characterized by their engagement in interprofessional interactions within the context of dentistry.

Within the smaller cohorts, specific scenarios, carefully formulated in accordance with the learning objectives set out in the scenario guidelines provided in the Supplementary Material 1, were distributed. Each group was tasked with deliberating upon the comprehensive set of healthcare professionals that would collectively manage the patient as per the given scenario. These scenarios were meticulously designed to facilitate peer-based experiential learning.

This iterative training approach was repeated twice, effectively engaging a cumulative total of 25 participants.

The objectives aimed to introduce participants to IPE, emphasize the benefits of collaborative teamwork in patient care, underscore the importance of working with diverse healthcare professionals, create a conducive environment for communication and teamwork, and simulate real-life patient care situations. Moreover, participants were guided to analyze clinical examination data, and to seek consultation or make referrals to specialized departments (dentistry, dietetics, nursing) as warranted.

The small group interactions revolved around pivotal inquiries, including:

- In the presented scenario, which cadre of healthcare professionals should collaboratively oversee the patient’s care? What substantiates these collective choices?
- What constitutes the patient’s principal grievance, concurrent health conditions, foreseeable risk factors, and which healthcare professionals should assume responsibility for mitigating these risks? How do these roles dovetail with that of the dentist?
- What advantages and obstacles underscore the endeavor of interprofessional cooperation?

The study highlights the significance of IPE in dental education, emphasizing its potential benefits in addressing patients’ special needs and improving overall healthcare outcomes.

### Statistical analysis

In order to assess the internal consistency of the data collected, Cronbach’s alpha coefficients were calculated for both pre- and post-SBPL programme responses. The analysis, performed at a significance level of *p* < 0.05, demonstrated commendable reliability across the investigated dimensions. The pre-SBPL programme responses yielded Cronbach’s alpha coefficients ranging from 0.518 to 0.922, with strong item-total correlations and squared multiple correlations ranging from 0.152 to 0.922. In parallel, the post-SBPL responses exhibited coefficients ranging from 0.096 to 0.937 for individual items, supported by robust sub dimensional reliabilities: Teams and collaboration (α = 0.916), Professional Identity (α = 0.915), and Roles and Responsibilities (α = 0.916). In particular, the scale’s overall reliability of the questionnaire, validated by a Cronbach’s alpha value of 0.915 for both phases, substantiates the internal consistency and robustness of the findings.

In order to comprehensively assess the impact of SBPL programme with a focus on IPE, the Wilcoxon Signed Rank Test was used to analyze the pre and post survey responses. Wilcoxon Signed Ranks Test was used to examine the differences between the averages obtained from the scale before and after the SBPL programme. The negative rank-based approach was used for calculations. Statistically significant differences were observed, as evidenced by the Z-scores (ranging from − 2.236 to -3.642) and associated two-tailed *p*-values (ranging from 0.000 to 0.025). These results highlight the significant impact of the SBPL intervention on variables related to teams and collaboration, professional identity, and roles and responsibilities in the context of IPE.

Data were organized within a Microsoft Excel file, and comprehensive analyses were performed using the Wilcoxon test, facilitated through SPSS 25.0 software. A predetermined significance level of *p* < 0.05 was adopted in this study.

## Results

This study was conducted with a total of 25 participants (*n* = 25). As the details can be seen below, 40% of the participants were women (*n* = 10), while 60% were men (*n* = 15). The mean age of the participants was 25 years (minimum: 21, maximum: 28) (Table 1).

**Table 1:** Participant demographics

Variable	Value	Frequency	Percentage	Variable	Value	Frequency	Percentage
Sex	female	10	40%	Age	21	1	4%
	male	15	60%		22	1	4%
	Total	25	100%		23	3	12%
Class	second class	1	4%		24	3	12%
	third class	2	8%		25	8	32%
	fourth class	4	16%		26	5	20%
	fifth class	9	36%		27	2	8%
	six class	9	36%		28	1	4%
	Total	25	100%		Total	25	100%
Completed the questionnaire before	yes	1	4%				
	no	24	96%				
	Total	25	100%				

Participating students from the countries shown in the graph above come from various universities across Europe including eastern, northern, southern and central Europe indicated in Table 2 below.

**Table 2:** Participants by institution and country

University/Dental School	Location	Frequency
University of Helsinki	Helsinki, Finland	3
Charité Faculty of Dentistry	Berlin, Germany	1
Lithuanian University of Health Science	Kaunas, Lithuania	1
Riga Stradins University	Riga, Latvia	1
European University of Skopje	Skopje, North Macedonia	1
Pavol Jozef Šafárik University	Kosice, Slovakia	1
Plovdiv Medical University	Plovdiv, Bulgaria	1
Carol Davila University of Medicine and Pharmacy	Bucharest, Romania	1
European University of Cyprus	Nicosia, Cyprus	2
Universita Degli Studi Padova	Padua, Italy	1
University of Ljubljana	Ljubljana, Slovenia	1
Marmara University	Istanbul, Türkiye	1
University of Belgrade	Belgrade, Serbia	1
University of Leeds	Leeds, United Kingdom	1
The University of Western Brittany	Brest, France	1
The Academic Centre for Dentistry Amsterdam (ACTA)	Amsterdam, Netherlands	1
Comenius University, Jessenius Medical Faculty	Bratislava, Slovakia	1
Julius Maximilians University	Würzburg, Germany	1
Universidad Europea De Madrid	Madrid, Spain	1
University of Athens	Athens, Greece	1
Charles University Faculty of Medicine in Pilsen	Pilsen, Czech Republic	1
University of Zagreb School of Dental Medicine	Zagreb, Croatia	1

As the participants were asked about the scale before the application, only 1 participant declared that he/she had filled out the RIPLS before. This participant was asked what interprofessional education is and what effect it has on their education. The answer given to the question was: *“IPE is a way for different health professionals to collaborate in an effective teamwork environment to provide a better and integrated health service where quality is assured.’’*.

The Table 3 above provides a whole overview of the entire scale, and each of the subscales exhibits a significantly diffrenece between pre and post results, encompassing 16 out of the 19 items (*p*<0.000). The preceding table presents the Cronbach’s Alpha values, affirming the substantial reliability of the findings. The mean values for each item, as well as those for the subscales and the overall scale, succinctly illustrate the impact of the SBPL programme in augmenting participants’ readiness (Table 2).

**Table 3:** Descriptive statistics of RIPLS items and subscales

	RIPLS Items	Pre			Post			*p* values
		Cronbach alpha	Mean	Std. Deviation	Cronbach alpha	Mean	Std. Deviation	
Teamwork and Collaboration	Item 1	0.904	4.60	0.50	0.917	4.88	0.33	0.008*
	Item 2	0.907	4.76	0.44	0.920	4.96	0.20	0.025*
	Item 3	0.904	4.52	0.51	0.914	4.84	0.47	0.011*
	Item 4	0.906	4.40	0.71	0.914	4.84	0.47	0.007*
	Item 5	0.903	4.48	0.69	0.916	4.84	0.47	0.021*
	Item 6	0.907	4.32	0.77	0.916	4.84	0.47	0.003*
	Item 7	0.904	4.24	0.72	0.918	4.92	0.28	0.001*
	Item 8	0.898	4.12	0.78	0.917	4.92	0.40	0.000*
	Item 9	0.905	4.68	0.48	0.916	4.88	0.44	0.096
Professional Identity	Item 10	0.906	4.08	0.86	0.916	4.68	0.70	0.002*
	Item 11	0.905	3.80	1.12	0.914	4.56	0.82	0.001*
	Item 12	0.917	3.80	1.08	0.943	3.40	1.41	0.239
	Item 13	0.910	4.04	1.06	0.919	4.88	0.33	0.000*
	Item 14	0.901	4.28	0.94	0.915	4.84	0.47	0.002*
	Item 15	0.911	4.20	1.00	0.919	4.84	0.37	0.003*
	Item 16	0.900	4.20	0.81	0.917	4.88	0.33	0.000*
Responsibilities and Roles	Item 17	0.901	4.32	0.85	0.915	4.88	0.44	0.004*
	Item 18	0.913	3.76	1.10	0.928	3.96	1.14	0.408
	Item 19	0.928	2.88	1.17	0.939	3.56	1.19	0.023*
	Teamwork and Collaboration Subscale	0.902	4.45	0.49	0.916	4.88	0.36	0.001*
	Professional Identity Subscale	0.901	4.05	0.63	0.915	4.58	0.45	0.000*
	Responsibilities and Roles Subscale	0.906	3.65	0.51	0.916	4.13	0.53	0.003*
	Total	0.901	4.18	0.47	0.915	4.65	0.38	0.000*

* *p* < 0,05

### Teamwork and collaboration subscale

Analyzing the results reveals a clear trend: among the 9 items, 8 show a significant difference, from pre to post subscale outcomes as seen in the Table 2 (*p* < 0.05). This indicates a notable shift in participants’ views on teamwork and collaboration following the programme. However, item 9 doesn’t exhibit a noticeable difference in responses (*p* > 0.05), showing that participants already recognized the importance of trust and respect before the intervention.

### Professional identity subscale

Out of the 7 items, 6 demonstrate a meaningful difference between pre-and post scale results (*p* < 0.05). However, item 12 doesn’t show any substantial variation between its pre- and post subscale responses (*p* > 0.05). This suggests that perceptions remained relatively constant for the item " Clinical problem solving can only be learnt effectively with students / professionals from my own school / organization.”

### Roles and responsibilities subscale

Items " Shared learning before and after qualification will help me become a better team worker " (item 17) and " I have to acquire much more knowledge and skill than other students / professionals in my own faculty / organization " (item 19) exhibit a significant change between pre- and post subscale results (*p* < 0.05). Conversely, there’s no significant difference between the pre- and post subscale results for the statement " I am not sure what my professional role will be / is " (item 18) (*p* > 0.05).

## Discussion

Our study targeted dental students studying in countries within the EHEA. Consequently, conducting the study on students attending the 70th EDSA meeting allowed us to easily access individuals with diverse demographic characteristics but resulted in a low participant count. The geographical and demographic diversity among our participants provided valuable insights into differing perspectives regarding readiness [24, 25]. Specifically focusing on individuals within Europe but with varying backgrounds and cultures was our primary aim. This deliberate selection aimed to be advantageous for examining readiness among students from different institutional cultures and holds potential benefits for future interprofessional collaboration we plan to undertake.

SBPL programme is an educational approach that leverages real-life scenarios to facilitate peer-to-peer learning among students in medical and dental education. This method involves students working together to analyze and solve problems based on realistic situations, allowing them to apply their knowledge and skills in a practical context [26]. Recognized as an effective strategy for promoting deeper learning and understanding, as it provides students with the opportunity to engage in active learning and critical thinking.

The scenarios we employed in our study aimed not to enhance professional knowledge and skills but to assess readiness in areas such as effective communication, teamwork, and a predisposition toward collaboration. Therefore, we do not perceive the varying educational years of the students as a constraint in our study. The limited number of participants is a recurrent challenge in IPE research, and it is essential to acknowledge this limitation in the scholarly article [27]. Addressing these aspects within our study underscores the importance of diverse participant backgrounds and underscores the necessity for larger sample sizes to achieve more comprehensive and representative outcomes within the realm of IPE readiness.

In the literature, it has been reported that compared to individuals with prior professional experience, the RIPLS might not yield consistent results. However, it shows potential for providing more dependable outcomes within a sample group who are either new to the profession or have not yet embarked on their professional journey. Consequently, this scale was selected for our study [28]. Additionally, the scale’s documented reliability among professionals in the medical and healthcare fields further justifies its selection [29, 30].

Traditionally, medical, and dental institutions hesitated to question educational methods or identify shortcomings. However, the demand for competency-based dental education necessitates integration of innovative teaching, contemporary technologies, and specialised IPE programs [22, 31]. This study addresses the evolving needs of dental education in response to global calls for enhanced interprofessional readiness [22, 23].

Employing a SBPL approach, our study aimed to problem solving experiences of interprofessional collaboration settings, supported by peer peer facilitators for effective engagement. Following SBPL session, participants exhibited significant improvements in terms of the readiness, demonstrating the effectiveness of the programme in improving teamwork and willingness to collaborate [32]. This is consistent with existing literature where IPE approach itself or similar proactive approaches has improved the collaborative skills of healthcare professionals [33]. Statistically, there is sufficient evidence to accept the hypothesis that the SBPL programme enhances undergraduate dental students’ readiness for IPE.

Based on the outcomes from the Teamwork and Cooperation Subscale, assessing students’ perspectives on collaborative learning and reciprocal respect, the SBPL programme demonstrated enhanced readiness across all items, except for the 9th item. Notably, there was no substantial alteration observed for item 9, emphasizing the importance of mutual respect and trust among students and professionals in small group settings. This lack of change could be attributed to the already elevated mean value of this item prior to the implementation of the program.

Similary, the absence of a notable difference in item 12, “Clinical problem solving can only be learned effectively with students/professionals in my own school/institution,” appears to be connected to the heightened awareness existing prior to the program [34].

Roles and responsibilities displayed comparatively modest changes compared to other sub-dimensions; nevertheless, the SBPL programme still contributed to a noteworthy improvement. This aligns with the program’s emphasis on roles, teamwork, and interprofessional collaboration.

Items related to potential role conflict or overlap didn’t show significant differences, suggesting that SBPL programme might not completely mitigate students’ concerns about their roles (Item 18). This highlights the intricacies of addressing professional roles in interprofessional contexts.

Post the intervention, noticeable changes were observed in the students’ attitudes toward collaborating with other disciplines, recognizing the necessity of cross-disciplinary learning, and acknowledging the importance of acquiring knowledge beyond their specialized domains. However, concerning the intricate realm of interprofessional collaboration, where the roles of health professionals can intermingle or potentially conflicting, the lack of significant change in related items (particularly Item 18) shows the ongoing need for IPE. Extending this approach to students from diverse health-related disciplines could potentially enhance the mitigation of these concerns.

The findings should be interpreted within the context of several limitations. The absence of a control group and reliance on a traditional pre-test and post-test comparison, coupled with a small sample size, influence the internal validity of the study. Given that participation in the course was voluntary, highly motivated students interested in IPE and patient safety were more likely to enroll, thereby leading to higher baseline values.

## Conclusions

Our study highlights the value of multidisciplinary perspectives and IPE within dental education. Within the limits of this study, demonstrates the effectiveness of a half-day SBPL programme with interprofessional scenarios in enhancing participants’ readiness. The programme increased dental students’ readiness to learn key cornerstones of IPE: teamwork and collaboration, professional identity, and roles and responsibilities. This study does not provide insight into the potential contributions of a comprehensive, long-term curriculum infused with IPE principles. This gap calls for further investigation into the sustained impact of IPE on the interprofessional competencies of dental school graduates.

In addition, future studies can be conducted to increase the number of participants and include not only dental students but also students from other health professions.

### Electronic supplementary material

Below is the link to the electronic supplementary material.

Supplementary Material 1

## Data Availability

The datasets used and/or analysed during the current study are available from the corresponding author on reasonable request.
