# Supplementary material for: Enhancing interprofessional education readiness in undergraduate dental students: a scenario-based peer learning programme
Source: BMC Oral Health. 2024 Jan 22;24:121. doi: 10.1186/s12903-024-03878-7 (PMC10801947; doi:10.1186/s12903-024-03878-7)
Supplement: Supplementary file 1 — Supplementary Material 1 [file 12903_2024_3878_MOESM1_ESM.docx]

**SCENARIO GUIDELINES FOR STUDY TITLED:** **ENHANCING INTERPROFESSIONAL EDUCATION READINESS IN UNDERGRADUATE DENTAL STUDENTS: A SCENARIO-BASED PEER LEARNING PROGRAMME**

**Learning Objectives:**

In the peer learning experience, it is aimed that the participants learn the skills of taking anamnesis related to their profession (questioning their complaints, reason for applying to the health institution, job, marital status, previous illnesses and surgeries, etc. in an appropriate language, showing the necessary respect and care) and examining the clinical examination data and consulting or directing you to an appropriate department (doctor, physiotherapist, psychologist, dietician, nurse).

It is aimed that the student will have effective communication skills, teamwork and cooperative attitude, and approach with a perspective that will reveal side effects, risks, preventive measures, complications related to general health and oral and dental health.

**Patient Personal Information**

**Patient Name and Surname:**

**Tel:**

**Address:**

**Date of Birth:**

**Basic Medical Problems (reason for consulting a physician):**

Scenario Short Narrative:

**Presentation:** General narrative of a few sentences, main complaint

**Clinical background:** History of illness, major operations, medication use, social position, family information

**Consultation:** Previous diagnoses and treatments, the patient's response to these treatments, the course of complaints over time

**Clinical issues:** Clinical Findings, vital signs, findings on diagnostic tools, persistent and improved symptoms

**Chronic condition management:** Knowledge of how the patient's chronic conditions, disabilities and other disadvantages have been handled so far.

**Mode of transfer:** patient's mode of transfer

**Debrief Supplementary File : SBPL programme scenarios were standardised according to the guidelines**

**Scenario-Based Peer Learning (SBPL) Programme Session Structure**

**Title of the Study:** Enhancing Interprofessional Education Readiness in Undergraduate Dental Students: A Scenario-Based Peer Learning Programme

**Date:** 22 August 2022

**Time:** 13.00-16.00

**Venue:** ADEMA Dental School, Palma De Mallorca, Spain

**Peer-Trainers:**

1. **Co-Trainer:** Ömer Faruk Sönmez, D.D.S., M.P.H. (University of Sheffield)
2. **Co-Trainer:** Catarina Silva, Pharm.D. (Lisbon University)

**Objectives:**

1. Introduce participants to the Interprofessional Education (IPE) concept.
2. Emphasize the potential benefits of collaborative teamwork in addressing patients with special needs, eliminating health inequalities, and improving overall healthcare outcomes.
3. Highlight the importance of working with diverse healthcare professionals to deliver comprehensive patient care.
4. Create a comfortable environment for communication and teamwork among students.
5. Simulate real-life patient care situations through scenarios, engaging participants in problem-solving exercises for collaboration and decision-making.
6. Peer-facilitate students to actively participate in group discussions, learn from each other, and present their findings to the group.

**Agenda:**

**I. Introduction (15 minutes)**

- Welcome and Icebreaker Activity
- Overview of the SBPL Session
- Brief Introduction of Trainers and Participants

**II. Orientation to Interprofessional Education (IPE) (20 minutes)**

- Presentation: Understanding Interproffesional Collaboration Settings
- Examples of Successful Interprofessional Collaboration
- Q&A Session

**III. Small Group Interactions with Scenarios (40 minutes)**

- Formation of Small Groups (5 individuals per group)
- Distribution of Scenarios
- Deliberations on Comprehensive Healthcare Professional Management
- Engagement with Peer Trainers for Guidance

**IV. Group Presentations (20 minutes)**

- Designated Spokesperson from Each Group Presents Findings
- Interaction with Other Groups and Peer Trainers
- Iterative Training Approach: Repeat with a Different Scenario

**V. Q&A and Group Discussion (15 minutes)**

- Open Floor for Questions and Comments
- Facilitated Group Discussion on Key Learnings
- Reflection on Interprofessional Collaboration

**VI. Conclusion and Debrief (10 minutes)**

- Summary of Key Takeaways
- Closing Remarks and Acknowledgments

**Debrief of Supplementary File: The objectives and the structure of SPBL programme is presented**
